# Diagnostic accuracy of supplemental three-dimensional breast ultrasound in the work-up of BI-RADS 0 screening recalls

**DOI:** 10.1186/s13244-024-01714-8

**Published:** 2024-05-31

**Authors:** Bianca M. den Dekker, Mireille J. M. Broeders, Carla Meeuwis, Wikke Setz-Pels, Alexander Venmans, Carla H. van Gils, Ruud M. Pijnappel

**Affiliations:** 1 Department of Radiology, University Medical Center Utrecht, Utrecht University, Utrecht, The Netherlands; 2 Department for Health Evidence, Radboud University Medical Center, Nijmegen, The Netherlands; 3 Dutch Expert Centre for Screening, Nijmegen, The Netherlands; 4 Department of Radiology, Rijnstate Hospital, Arnhem, The Netherlands; 5 Department of Radiology, Catharina Hospital, Eindhoven, The Netherlands; 6 Department of Radiology, Elisabeth-TweeSteden Hospital, Tilburg, The Netherlands; 7 Julius Center for Health Sciences and Primary Care, University Medical Center Utrecht, Utrecht University, Utrecht, The Netherlands

**Keywords:** Breast cancer, Automated breast ultrasound, BI-RADS 0

## Abstract

**Objective:**

To evaluate the diagnostic accuracy of supplemental 3D automated breast ultrasound (ABUS) in the diagnostic work-up of BI-RADS 0 recalls. We hypothesized that 3D ABUS may reduce the benign biopsy rate.

**Materials and methods:**

In this prospective multicenter diagnostic study, screening participants recalled after a BI-RADS 0 result underwent bilateral 3D ABUS supplemental to usual care: digital breast tomosynthesis (DBT) and targeted hand-held ultrasound (HHUS). Sensitivity, specificity, positive predictive value, and negative predictive value of 3D ABUS, and DBT plus HHUS, were calculated. New 3D ABUS findings and changes of management (biopsy or additional imaging) were recorded.

**Results:**

A total of 501 women (median age 55 years, IQR [51–64]) with 525 BI-RADS 0 lesions were included between April 2018 and March 2020. Cancer was diagnosed in 45 patients. 3D ABUS sensitivity was 72.1% (95% CI [57.2–83.4%]), specificity 84.4% (95% CI [80.8–87.4%]), PPV 29.2% (95% CI [21.4–38.5%]), and NPV 97.1% 95.0–98.4%). Sensitivity of DBT plus HHUS was 100% (95% CI [90.2–100%]), specificity 71.4% (95% CI [67.2–75.2%]), PPV 23.8% (95% CI [18.1–30.5%]) and NPV 100% (95% CI [98.7–100%]). Twelve out of 43 (27.9%) malignancies in BI-RADS 0 lesions were missed on 3D ABUS, despite being detected on DBT and/or HHUS. Supplemental 3D ABUS resulted in the detection of 57 new lesions and six extra biopsy procedures, all were benign.

**Conclusion:**

3D ABUS in the diagnostic work-up of BI-RADS 0 recalls may miss over a quarter of cancers detected with HHUS and/or DBT and should not be used to omit biopsy. Supplemental 3D ABUS increases the benign biopsy rate.

**Trial registration:**

Dutch Trial Register, available via https://www.onderzoekmetmensen.nl/en/trial/29659

**Critical relevance statement:**

Supplemental 3D automated breast ultrasound in the work-up of BI-RADS 0 recalls may miss over a quarter of cancers detected with other methods and should not be used to omit biopsy; ABUS findings did increase benign biopsy rate.

**Key Points:**

- Automated breast ultrasound (ABUS) may miss over 25% of cancers detectable by alternative methods.
- Don’t rely solely on 3D ABUS to assess indication for biopsy.
- New findings with supplemental 3D ABUS increase the benign biopsy rate.

**Graphical Abstract:**

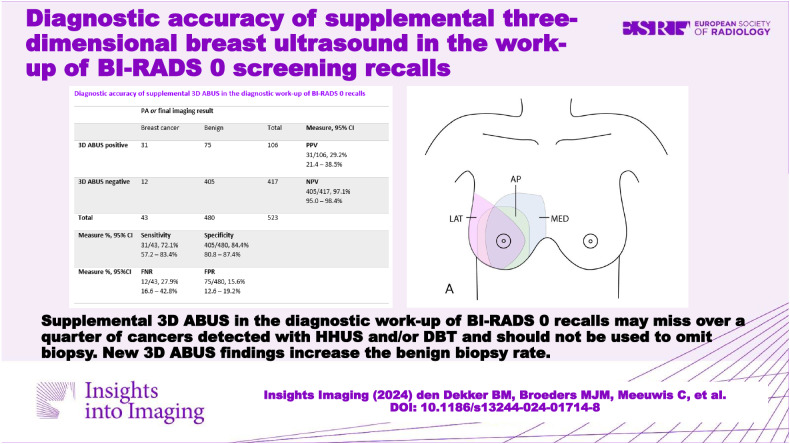

## Introduction

In the Netherlands, women between the ages of 50 and 75 years are invited for biennial breast cancer screening using full-field digital mammography. Each year approximately one million women participate and ~23 per 1000 participants are recalled and referred to a hospital for diagnostic work-up of inconclusive (BI-RADS 0) or suspicious findings (BI-RADS 4/5) [1]. The BI-RADS 0 recalls account for the largest share of all recalls with approximately 11.000/year. Of these women, 25–30% undergo breast biopsy and 10–12% are diagnosed with breast cancer [1]. Therefore, the BI-RADS 0 recalls have an important impact on the false-positive recall rate and benign biopsy rate. We hypothesized that supplemental three-dimensional automated breast ultrasound (3D ABUS) in the diagnostic work-up of BI-RADS 0 recalls may reduce the benign biopsy rate.

3D ABUS was originally designed as an adjunct screening tool for women with dense breasts to overcome the limitations of hand-held ultrasound (HHUS), which is operator-dependent, lacks standardization and reproducibility, and imposes a considerable workload on radiologists [2]. 3D ABUS enables standardized acquisition of volumetric images of the whole breast, facilitating double reading and objective comparison with previous imaging. The diagnostic accuracy of 3D ABUS has been reported to be comparable to HHUS [3–9]. A meta-analysis by Meng et al showed a pooled sensitivity of ABUS of 92% (range: 89.9–93.8%) and specificity of 84.9% (range 82.4–87%), with no significant difference in diagnostic accuracy between HHUS and ABUS. Three-dimensional reconstruction of the ABUS imaging enables evaluation in the coronal plane, which has been reported to significantly aid in the differentiation between benign and malignant breast lesions [10]. Spiculation of malignant lesions and the retraction phenomenon caused by invasive growth and surrounding desmoplastic reaction lead to architectural distortions, which may be appreciated best in the coronal plane [7, 11, 12]. Using the coronal view, benign lesions seen in the transverse plane may be downgraded in up to 18% of benign cases, potentially avoiding benign biopsy [10]. False positive findings necessitate further imaging, biopsy, or additional follow-up examinations which often cause anxiety in the patient, increase healthcare costs, and sometimes lead to biopsy-related morbidity. 3D ABUS in the diagnostic work-up of BI-RADS 0 recalls may reduce the benign biopsy rate. The objective of this study was to evaluate the diagnostic accuracy of supplemental 3D ABUS in the diagnostic work-up of Dutch breast cancer screening participants recalled with a BI-RADS 0 screening mammography result.

## Materials and methods

This prospective multicenter diagnostic study was approved by the Medical Research Ethics Committee of the University Medical Center Utrecht. Written informed consent was obtained from all participants prior to the 3D ABUS examination. The study protocol is available via Dutch Trial Register (https://www.onderzoekmetmensen.nl/en/trial/29659).

**Fig. 1:**
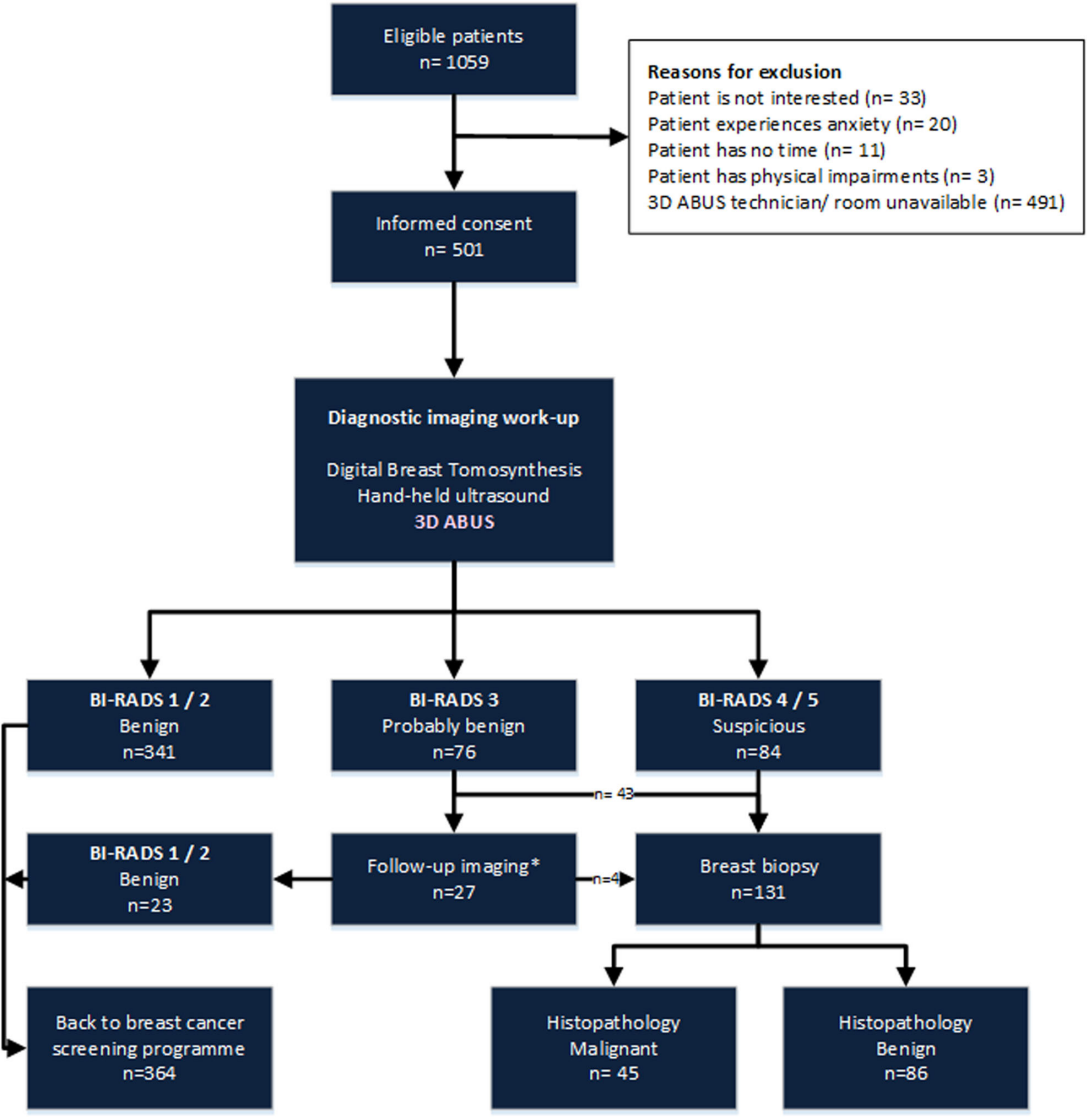
Study flowchart. A total of 1059 women, recalled for diagnostic work-up after a BI-RADS 0 screening mammography result and referred to one of three participating hospitals between April 2018 and March 2020 were considered eligible. A total of 501 women signed informed consent and received supplemental 3D ABUS, in addition to usual care diagnostic work-up with DBT and HHUS. All breast imaging (DBT, HHUS and 3D ABUS) was evaluated to assign each patient a BI-RADS score. 341 participants were classified BI-RADS 1 or 2 (benign); these participants were referred back to the breast cancer screening programme. 76 participants were classified BI-RADS 3; of these women, 43 underwent breast biopsy and 33 were invited for follow-up imaging. Six participants were lost to follow up. After a minimum of six months, follow-up imaging was performed in 27 participants, after which 23 were referred back to the screening programme and four underwent breast biopsy. 84 patients were classified BIRADS 4/5; all underwent breast biopsy. In total, 45/501 participants were diagnosed with cancer; 44 had breast cancer and one participant was diagnosed with non-Hodgkin lymphoma after detection of an intramammary lymph node on breast imaging

Between April 2018 and March 2020, all Dutch breast cancer screening participants (aged 50–75 years), recalled for diagnostic work-up after a BI-RADS 0 screening mammography result and referred to one of three participating non-academic hospitals, were considered eligible for participation (Fig. 1). Women who were unable or unwilling to provide informed consent were excluded. Reasons for non-participation were recorded. The conventional diagnostic work-up (usual care) consisted of mediolateral oblique (MLO) and craniocaudal (CC) digital breast tomosynthesis (DBT) of both breasts and targeted hand-held ultrasound (HHUS) of the BI-RADS 0 lesion. DBT was performed on Hologic Dimensions, Hologic Dimensions 3D, or Hologic Selenia Dimensions. HHUS was performed on a Philips Epiq 7 G or Toshiba Aplio 500 system. In line with clinical practice, DBT and HHUS were evaluated together leading to a final BI-RADS classification. In addition to the usual care work-up, all study participants received supplemental bilateral 3D ABUS. 3D ABUS imaging was evaluated directly after the evaluation of DBT and HHUS by the same radiologist, who was not blinded for the results of previous imaging.

All breast imaging was interpreted according to the ACR BI-RADS Atlas (Fifth Edition) by one of 15 radiologists (including C.M., W.S.P., and A.V.), all dedicated breast radiologists whose experience ranged from 1.5 to 29 years. BI-RADS classification of DBT plus HHUS and 3D ABUS, additional findings with 3D ABUS, changes in management after 3D ABUS (biopsy or additional imaging), and adverse events were recorded.

Breast density was visually assessed on reconstructed DBT and classified into four categories according to the ACR BI-RADS Atlas (Fifth Edition): almost entirely fatty breasts (A), scattered areas of fibroglandular density (B), heterogeneously dense breasts (C), and extremely dense breasts (D) [13]. Malignant lesion size, measured as the largest diameter on any imaging modality, was recorded.

### 3D ABUS imaging

**Fig. 2:**
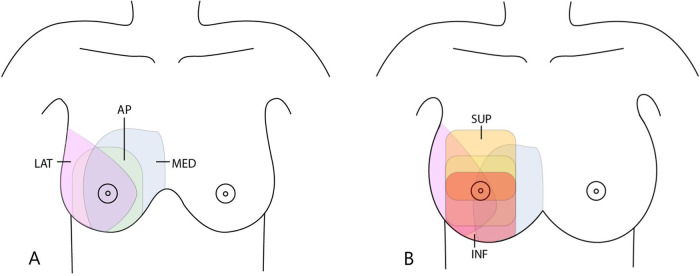
Ultrasound views of the breast obtained with 3D ABUS examination. **A** The three common views: anterior-posterior (AP), lateral (LAT) and medial (MED). **B** The five views for full coverage of large breasts with additional superior (SUP) and inferior (INF) views

3D ABUS imaging was obtained by trained technicians using an ABUS system (Invenia^TM^ ABUS, Automated Breast Ultrasound System, GE Healthcare, Sunnyvale, CA, USA). This ABUS system consists of a scan station, equipped with a 6–15 MHz wide transducer attached to a flexible arm, a touch-screen monitor, and a dedicated workstation for image evaluation. All technicians received a three-day training in 3D ABUS examination from a GE application specialist before the start of the study and a refresher course after approximately six months. Patients were scanned in supine position with the ipsilateral arm raised above the head. A lotion was applied to the breast to establish good skin contact. In each scan a volume data set up to 16.9 × 15.3 × 5.0 cm was obtained with slice intervals of 2 mm. Of each breast, anterior-posterior, lateral, and medial views were obtained. Additional superior and inferior anterior-posterior views were obtained in large breasts (Fig. 2).

3D ABUS imaging was sent to the dedicated workstation for multiplanar reconstruction and review in the transverse, sagittal, and coronal plane. All radiologists received five hours of peer-to-peer training in 3D ABUS interpretation from an expert radiologist and had access to a digital learning environment to practice 3D ABUS readings.

Breast cancer diagnosis was defined as a histopathological diagnosis of DCIS or invasive malignancy, all other histopathological findings were considered benign. Histopathology was indicated for all lesions classified BI-RADS 4 or 5. In cases with no indication for histopathological diagnosis, the final conclusion after assessment of all available breast imaging was used. A final BI-RADS 1 or 2 category was considered benign. All women with a final BI-RADS 1 or 2 result were referred back to the breast cancer screening programme to get reinvited for screening mammography in the next round after two years. In case of a BI-RADS 3 result after diagnostic work-up, the Dutch guideline recommends tissue diagnosis or short-interval (i.e., six months) follow-up. In all participants with a BI-RADS 3 result without histopathological diagnosis, the results of follow-up imaging (and histopathology if available) of at least six months after inclusion were recorded.

### Statistical analysis

Data was analyzed using SPSS (IBM® SPSS Statistics® version 25) and RStudio (R version 3.6.1). Diagnostic accuracy was calculated for 3D ABUS and for DBT *plus* HHUS. Imaging examinations were considered positive if the BI-RADS 0 lesion of referral was categorized as BI-RADS ¾/5 and negative if categorized as BI-RADS ½. Sensitivity was calculated by dividing the number of true-positive examinations by the total number of breast cancer cases. Specificity was calculated by dividing the number of true-negative examinations by the total number of benign cases. Positive predictive value was calculated by dividing the number of true-positive examinations by the total number of positive examinations. The negative predictive value was calculated by dividing the number of true-negative examinations by the total number of negative examinations. 95% confidence intervals were calculated using the Agresti-Coull method [14].

## Results

### Study population

A total of 1059 women, recalled for diagnostic work-up after a BI-RADS 0 screening mammography result and referred to one of three participating hospitals between April 2018 and March 2020 were considered eligible. A total of 501 women (median age 55 years, IQR [51–64]) signed informed consent (Fig. 1). Recorded reasons for patient non-participation were: no interest in research participation (*n* = 33), breast cancer anxiety related to the diagnostic work-up (*n* = 20), no time for the ABUS examination (*n* = 11) and three women could not participate because of physical impairments that hampered correct positioning for 3D ABUS scanning. Most women (*n* = 491) were excluded because there was no study technician, study radiologist, or ultrasound room available at the time of diagnostic work-up.

From the total of 501 study participants, ten women were recalled for a BI-RADS 0 result of both breasts, twelve had two BI-RADS 0 lesions in one breast, and one woman was recalled with three BI-RADS 0 lesions, resulting in a total number of 525 screen-detected BI-RADS 0 lesions. The majority of the lesions were described as masses (327/525) on screening mammography, followed by asymmetries (159/525) and architectural distortions in one direction (24/525). There were 15 lesions categorized as BI-RADS 0 that, in hindsight, should have been categorized differently based on the lesion description in the recall letter: eleven architectural distortions in two directions, two masses with architectural distortion, one calcifications-only lesion and one mass with calcifications. According to the Dutch screening guidelines the mass with calcifications should have been classified as BI-RADS 5, and the other lesions as BI-RADS 4. However, as they were BI-RADS 0 recalls and met all inclusion criteria, these cases were included in our analysis.

Out of 501 participants, 78 women (15.6%) had almost entirely fatty breasts, 320 (63.9%) had scattered areas of fibroglandular density, 93 (18.6%) had heterogeneously dense breasts and ten (2%) had extremely dense breasts.

### Diagnostic work-up results and histopathology

After diagnostic work-up, including DBT, HHUS and 3D ABUS, the final clinical BI-RADS classification was BI-RADS 1 or 2 in 341 (68.1%) participants; these participants were referred back to the breast cancer screening programme. 12 (2.4%) participants with a final BI-RADS 5 and 72 (14.4%) with a BI-RADS 4 result underwent breast biopsy for histopathological diagnosis, yielding breast cancer in 12/12 (100%) and in 29/72 (40.3%) participants respectively. Out of 76 (15.2%) participants with a BI-RADS 3 result, 43 women underwent breast biopsy and 33 were invited for follow-up imaging. After a minimum of six months, follow-up imaging was performed in 27 participants and six patients were lost to follow-up. Based on all available breast imaging for these six patients they were considered benign in further analysis. After follow-up imaging 23/27 were referred back to the screening programme and four underwent breast biopsy. Out of 76 participants with a final BI-RADS 3 result, three (3.9%) were diagnosed with breast cancer. In total, 44/501 (8.8%) participants were diagnosed with breast cancer (median lesion size 9.5 mm, range 4–69 mm). In addition, one participant was diagnosed with non-Hodgkin lymphoma after the detection of an intramammary lymph node on breast imaging. Out of all 45 detected cancers, 43 resulted from a BI-RADS 0 lesion, and two resulted from a new lesion detected during diagnostic work-up on HHUS and DBT. Histopathological results are shown in Table 1.

**Table 1:** Results of histopathological biopsy in 133 patients

Result	Biopsy in work-up *n* = 121	Biopsy after follow-up *n* = 3	Biopsy new lesions *n* = 9
Malignant	*n* = 42	*n* = 1	*n* = 2
Invasive carcinoma NST	28	1	1
Invasive lobular carcinoma	6		1
Tubular carcinoma	1		
Mucinous carcinoma	2		
Invasive micropapillary carcinoma	2		
DCIS	2		
B-cel non-Hodgkin lymphoma	1		
High risk	*n* = 14	NA	
Intraductal papilloma/papillary lesion	8		
Complex sclerosing lesion/radial scar	3		
FEA/CCL	3		
Benign	*n* = 64	*n* = 2	*n* = 7
Fibrosis/fibrocystic changes	22	1	3
Fibroadenoma	14	1	1
Reactive changes	6		
UDH	5		1
Lymph node	3		
Hemangioma	2		
PASH	2		
Apocrine cyst/metaplasia	4		
Adenosis	1		
Benign skin lesion	1		1
No abnormalities	4		

*CCL* columnar cell lesion, *DCIS* ductal carcinoma in situ, *FEA* flat epithelial atypia, *NA* not applicable, *NST* no special type, *PASH* pseudo angiomatous stromal hyperplasia, *UDH* usual-type ductal hyperplasia

### Diagnostic accuracy of 3D ABUS

**Fig. 3:**
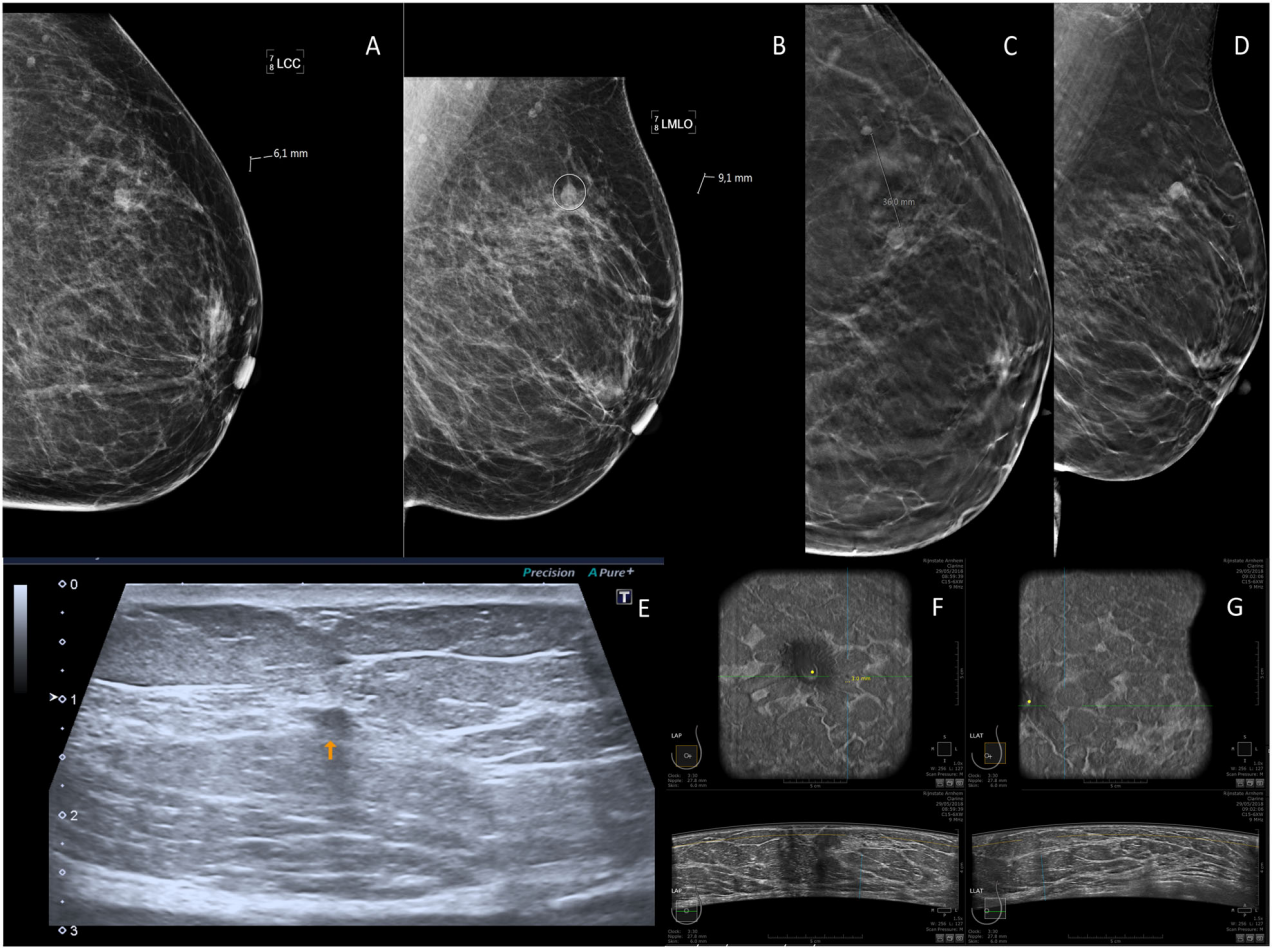
Example of a breast cancer missed on 3D ABUS. A 60-year old woman was recalled after detection of a new mass in the superior lateral quadrant of the left breast, classified as BI-RADS 0 on screening mammography (**A**: CC view, **B**: MLO view). The lesion was classified as BI-RADS 4 on digital breast tomosynthesis (**C**: CC view, **D**: MLO view). On hand-held ultrasound the lesion was difficult to visualize and described as a mass of 4 mm in diameter, classified as BI-RADS 3 (**E**: ultrasound). The lesion was missed on 3D ABUS imaging. In hindsight, the exact location of the lesion was reevaluated on 3D ABUS and a 3 mm lesion was noted on the anterior-posterior view (**F**: AP view, **G**: lateral view). The patient underwent biopsy (PA: invasive carcinoma NST), followed by a radioactive seed-guided lumpectomy. Histopathology showed a grade I invasive carcinoma NST, ER+/PR+/HER2− with a diameter of 4 mm, TNM stage (8th edition): pT1aN0(i-)(sn)

Out of the 525 BI-RADS 0 lesions, 42 yielded breast cancer and one resulted in the diagnosis of non-Hodgkin lymphoma. Complete 3D ABUS examination was available for 523/525 BI-RADS 0 lesions (Table 2), two examinations were terminated before completion due to adverse events. 3D ABUS sensitivity was 72.1% (95% CI [57.2–83.4%]), specificity was 84.4% (95% CI [80.8–87.4%]), FPR was 15.6% (95% CI [12.6–19.2%]), FNR was 27.9% (16.6–42.8%), PPV was 29.2% (95% CI [21.4–38.5%]) and NPV was 97.1% (95.0–98.4%) (Table 2). Twelve lesions recalled from screening with a BI-RADS 0 result that contained cancer were missed on 3D ABUS despite being detected on DBT and/or HHUS. Sensitivity of DBT plus HHUS was 100% (95% CI [90.2–100%]), specificity was 71.4% (95% CI [67.2–75.2%]), PPV was 23.8% (95% CI [18.1–30.5%]), and NPV was 100% (95% CI [98.7–100%]) (Table 2). The cancers missed with 3D ABUS included eight cases of grade I invasive carcinoma (size range 2–18 mm), one grade II invasive carcinoma (4 mm), one grade II invasive lobular carcinoma (9 mm), one grade II invasive micropapillary carcinoma (5 mm) and one grade I DCIS (5 mm). An imaging example of a breast cancer missed on 3D ABUS is provided in Fig. 3.

In addition, there were two new cancers detected on both DBT and HHUS during diagnostic work-up that was missed on 3D ABUS. Cancers that were missed on 3D ABUS had a mean lesion size of 9.0 mm, versus 14.6 mm in cancers detected on 3D ABUS (*p*-value 0.06). A detailed overview of all 3D ABUS missed breast cancers is presented in Table 3.

**Table 2:** Measures of diagnostic accuracy

	PA or final imaging result			
	Breast cancer	Benign	Total	Measure % (95% CI)
3D ABUS				
3D ABUS positive	31	75	106	PPV 31/106, 29.2% 21.4–38.5%
3D ABUS negative	12	405	417	NPV 405/417, 97.1% 95.0–98.4%
Total	43	480	523	
Measure %, 95% CI	Sensitivity 31/43, 72.1% 57.2–83.4%	Specificity 405/480, 84.4% 80.8–87.4%		
Measure %, 95% CI	FNR 12/43, 27.9% 16.6–42.8%	FPR 75/480, 15.6% 12.6–19.2%		

	PA or final imaging result			
	Breast cancer	Benign	Total	Measure % (95% CI)
DBT plus HHUS				
HHUS + DBT positive	43	138	181	PPV 43/181, 23.8% 21.4–38.5%
HHUS + DBT negative	0	344	344	NPV 344/344, 100% 95.0–98.4%
Total	43	482	525	
Measure %, 95% CI	Sensitivity 43/43, 100% 90.2–100%	Specificity 344/482, 71.4% 67.2–75.2%		
Measure %, 95% CI	FNR 0/43, 0% 0–9.8%	FPR 138/482, 28.6% 24.8–32.8%		

Breast cancer diagnosis was defined as a histopathological diagnosis of DCIS or invasive malignancy, all other histopathological findings were considered benign. Histopathology was indicated for all lesions classified BI-RADS 4 or 5. In case histopathology was not available, the final conclusion after assessment of all available breast imaging was used. Imaging examinations were considered positive if the BI-RADS 0 lesion of referral was categorized as BI-RADS 3/4/5 and negative if categorized as BI-RADS 1/2. For BI-RADS 3 lesions without histopathological diagnosis, the results of follow-up imaging (and histopathology if available) of at least six months after inclusion were taken into account

*3D ABUS* three-dimensional automated breast ultrasound system, *DBT* digital breast tomosynthesis, *HHUS* hand-held ultrasound, *FPR* False-positive rate, *FNR* false-negative rate, *NPV* negative predictive value, *PPV* positive predictive value

**Table 3:** Overview of breast cancers missed on 3D ABUS

	Age	Breast density	Screening mammography	Digital breast tomosynthesis	Hand-held ultrasound	3D ABUS	Histopathology
1	68	B	BI-RADS 0 right breast, asymmetry inferior breast on MLO view	BI-RADS 4 right breast, spiculated irregular mass 6 mm	BI-RADS 4 right breast, 1. spiculated mass 9 mm 2. subareolar architectural distortion with calcifications	BI-RADS 4 right breast, 1. lesion is not visualized 2. subareolar architectural distortion	1. Invasive carcinoma NST, grade I ER + /PR + /HER2 −, 2 mm 2. DCIS, grade II, 80 mm
2	52	B	BI-RADS 0 right breast, asymmetry superior lateral quadrant	BI-RADS 4 right breast, spiculated irregular mass	BI-RADS 4 right breast, spiculated irregular mass	BI-RADS 1 right breast lesion is not visualized	Invasive carcinoma NST, grade I ER + /PR − /HER2 − 12 mm
3	64	A	BI-RADS 0 right breast, mass superior lateral quadrant	BI-RADS 5 right breast, spiculated irregular mass, 5 mm	BI-RADS 5 right breast, spiculated mass, 5 mm	BI-RADS 1 right breast, lesion is not visualized	Invasive micropapillary carcinoma, grade II, ER + /PR + /HER2 − 5 mm
4	63	B	BI-RADS 0 right breast, mass superior lateral quadrant	BI-RADS 4 right breast bilobar mass indistinct margin, 10 mm	BI-RADS 1 right breast lesion is not visualized	BI-RADS 1 right breast, lesion is not visualized	DCIS, grade I 5 mm
5	55	C	BI-RADS 0 left breast, asymmetry superior breast on MLO view	BI-RADS 3 left breast, asymmetry 7 mm	BI-RADS 3 left breast, dubious mass	BI-RADS 1 left breast, lesion is not visualized	Invasive carcinoma NST, grade I ER + /PR − /HER2 − 8 mm
6	52	B	BI-RADS 0 left breast, asymmetry superior breast on MLO view	BI-RADS 4 left breast, irregular mass superior lateral quadrant 5 mm	BI-RADS 4 left breast, spiculated mass 5 mm	BI-RADS 1 left breast, lesion is not visualized	Invasive carcinoma NST, grade I ER + /PR + /HER2 − 5 mm and DCIS grade I
7	56	B	BI-RADS 0 left breast, mass medial central breast	BI-RADS 3 left breast, global asymmetry medial central	BI-RADS 4 left breast, irregular mass 6 mm	BI-RADS 2 left breast, lesion is not visualized	Invasive lobular carcinoma, grade II ER + /PR + /HER2 − 9 mm
8	52	B	BI-RADS 0 left breast, mass central superior breast	BI-RADS 3 left breast, irregular mas 4 mm	BI-RADS 4 left breast, 1. spiculated mass 5 mm 2. lobulated mass 13 mm (BI-RADS 3)	BI-RADS 3 left breast, 1. Spiculated mass is not visualized 2. Lobulated mass 13 mm	1. Invasive carcinoma NST, grade I ER + /PR −/HER2 − 6 mm 2. Fibroadenoma
9	60	B	BI-RADS 0 left breast, mass superior lateral quadrant	BI-RADS 4 left breast, mass indistinct margin 8 mm	BI-RADS 3 left breast, dubious mass 4 mm	BI-RADS 1 left breast, lesion is not visualized	Invasive carcinoma NST, grade I ER + /PR + /HER2 − 4 mm
10	71	B	BI-RADS 0 left breast, mass superior medial quadrant	BI-RADS 4 left breast, spiculated mass 6 mm	BI-RADS 4 left breast, spiculated mass 5 mm	BI-RADS 1 left breast, lesion is not visualized	Invasive carcinoma NST, grade II ER + /PR + /HER2 − 4 mm
11	60	B	BI-RADS 0 right breast, architectural distortion on CC view	BI-RADS 4 right breast, 11 mm	BI-RADS 4 right breast, 7 mm	BI-RADS 1 right breast, lesion is not visualized	Invasive carcinoma NST grade I ER + /PR −/HER2 − 10 mm and DCIS grade II 10 mm
12	69	B	BI-RADS 0 left breast, mass superior central breast	BI-RADS 4 left breast, irregular mass 21 mm	BI-RADS 4 left breast, irregular mass 19 mm	BI-RADS 2 left breast, lesion is not visualized	Invasive carcinoma NST, grade I, ER + /PR + /HER2 − 18 mm
13*	55	A	BI-RADS 0 right breast, asymmetry medial breast on CC view	BI-RADS 4 right breast, 1. mass 6 mm (BI-RADS 3) 2. linear fine pleomorphic calcifications 25 mm	BI-RADS 4 right breast, 1. mass 4 mm (BI-RADS 3) 2. linear fine pleomorphic calcifications 20 mm	BI-RADS 3 right breast, 1. mass 2. lesion is not visualized	1. Invasive carcinoma NST, grade II ER + /PR + /HER2 − 6 mm 2. DCIS grade III, 40 mm
14*	54	A	BI-RADS 0 right breast, mass inferior medial quadrant	BI-RADS 4 right breast, 1. circumscript mass 12 mm 2. microlobulated mass 10 mm	BI-RADS 4 right breast, 1. circumscript mass 13 mm 2. microlobulated mass 11 mm	BI-RADS 4 right breast, 1. mass 2. lesion is not visualized	1. Invasive carcinoma NST, grade I ER + /PR + /HER2 − 8 mm with DCIS grade II 18 mm 2. Invasive carcinoma NST, grade I ER + /PR + /HER2 − 18 mm and DCIS grade II

* Two breast cancers missed on 3D ABUS resulted from a new lesion detected on DBT and HHUS during diagnostic work-up

### New 3D ABUS findings and changes of management

Supplemental 3D ABUS resulted in the detection of 57 new lesions, which were all benign (Table 4). There was one new lesion classified BI-RADS 4 on 3D ABUS for which an extra target HHUS was performed, at which the lesion was classified as benign. There were six new BI-RADS 3 lesions; in five a target HHUS and biopsy were performed, yielding benign histopathological diagnoses in all. In the remaining BI-RADS 3 lesion a follow-up HHUS was performed and the lesion was classified as BI-RADS 2.

Furthermore, one BI-RADS 0 lesion was classified BI-RADS 2 on DBT and HHUS, but BI-RADS 3 on 3D ABUS, leading to an additional target HHUS and biopsy procedure. In total, 3D ABUS resulted in six extra benign biopsy procedures.

**Table 4:** Overview of new lesions detected on 3D ABUS

New 3D ABUS lesions	*n* (%) *n* = 57	Management consequences *n* = 7	Extra biopsy *n* = 5
BI-RADS 4	1 (1.8%)	1 extra target HHUS: BI-RADS 2	NA
BI-RADS 3	6 (10.9%)	5 extra target HHUS + biopsy 1 follow-up HHUS: BI-RADS 2	5, histopathology all benign
BI-RADS 2			
Cyst(s)	37 (67.3%)	NA	NA
Intramammary lymph node	5 (9.1%)		
Ductectasia	3 (5.5%)		
Lipoma(s)	2 (3.6%)		
Fibroadenoma	1 (1.8%)		

*HHUS* hand-held ultrasound, *NA* not applicable

### Adverse events

In two participants an erythematous skin reaction occurred after application of the lotion. In one participant the 3D ABUS examination was not completed for this reason. In both patients, the skin redness resolved spontaneously within 48 h. One participant experienced pain due to breast compression during the 3D ABUS examination and the examination was prematurely terminated.

## Discussion

This multicenter diagnostic study evaluated the diagnostic accuracy of 3D ABUS, supplemental to DBT and HHUS, in 501 women recalled after a BI-RADS 0 screening mammography. 3D ABUS sensitivity was 72.1% (95% CI [57.2–83.4%]), and specificity 84.4% (95% CI [80.8–87.4%]). On 3D ABUS 12 out of 43 malignancies in BI-RADS 0 lesions were missed, despite being detected on DBT or HHUS. In contrast to our hypothesis that 3D ABUS may reduce the benign biopsy rate, these findings indicate that 3D ABUS should not be used to omit biopsy. Supplemental 3D ABUS in addition to HHUS and DBT resulted in the detection of 57 new lesions and six extra biopsy procedures, all of which were benign. As such, supplemental 3D ABUS increased the benign biopsy rate.

Previous studies yielded a better diagnostic performance of 3D ABUS compared to our study, although differences in study populations limit comparability. Hellgren et al found a sensitivity of 88% and specificity of 89.2% of 3D ABUS in 113 women recalled because of a suspicious mammographic finding in screening [15]. However, this population was not limited to BI-RADS 0 recalls and included lesions with a higher suspicion of malignancy (further progressed state of disease), which may explain the higher sensitivity compared to our study. Several other studies, performed in even more heterogeneous study populations, found a 3D ABUS sensitivity ranging from 74% to 100%, and specificity of 68–95% [4, 7, 8, 11, 16, 17].

In our study, cancers that were missed on 3D ABUS had a mean lesion size of 9.0 mm versus 14.6 mm in cancers that were detected on 3D ABUS. Radiologists commented that the resolution of 3D ABUS was lower compared to HHUS, which complicated lesion detection and characterization, specifically of smaller lesions. In line with this, Shin et al described that lesion detection was reliable only when the mean lesion diameter was > 1.2 cm and Jeh et al mentioned that smaller lesions were statistically significantly less frequently detected on 3D ABUS [18, 19]. In our study, there were nine cancers > 1.2 cm, of which one, lesion size 2.2 cm, was missed on 3D ABUS.

When interpreting the measures of diagnostic accuracy it is important to recognize the learning curve of 3D ABUS readings. Many studies do not report the experience of 3D ABUS readers and in the literature, there is no consensus on the number of examinations that a radiologist must have interpreted to obtain the right skill level. As with other imaging modalities, experience improves the performance of radiologists. In our study 15 study radiologists participated, resulting in an average of ~33 ABUS examinations per radiologist. Although all radiologists received training and were encouraged to practice 3D ABUS readings in a digital learning environment, the limited experience with 3D ABUS and lack of continuity, the number of examinations may have been insufficient to reach the optimal skill level. In addition, for technicians, there is a learning curve to obtain state-of-the-art imaging with adequate breast compression and full breast coverage [20]. In our study, all technicians and radiologists were equally (in)experienced in obtaining/evaluating 3D ABUS imaging and the potential impact of the level of experience on the lesions missed in 3D ABUS could not be evaluated.

An important limitation of our study was that radiologists assessed the 3D ABUS imaging directly after evaluation of the DBT and HHUS. With unblinded sequential evaluation, the radiologist may be more inclined to score the 3D ABUS in line with the results of previous imaging. However, despite the detection of a suspicious lesion on DBT and/or HHUS, in over a quarter of cancers in BI-RADS 0 recalls the lesion was missed on 3D ABUS. This further underlines that 3D ABUS should not be used to omit biopsy.

Follow-up imaging was lacking in 6 out of 33 patients scheduled for follow-up after a BI-RADS 3 result. The six lesions were considered breast cancer negative based on all available imaging and included in our analysis. Exclusion of these six lesions does not affect the reported 3D ABUS sensitivity (31/43, 72.1%) nor the increase in benign biopsies.

Looking to the future of 3D ABUS, development should focus on further improvement of 3D ABUS resolution. Future research may look into stratified analysis for breast density and different applications of 3D ABUS, such as operative planning and follow-up of benign lesions [20, 21]. In addition, prototypes are developed that combine ABUS and DBT in a single device without decompression of the breast, offering practical and logistic advantages [22]. Furthermore, researchers and clinicians adopting 3D ABUS should be aware of the learning curve regarding image interpretation and acquisition.

In conclusion, 3D ABUS in the diagnostic work-up of breast cancer screening participants recalled with a BI-RADS 0 result may miss over a quarter of cancers detected on HHUS and/or DBT and should not be used to omit biopsy. Supplemental 3D ABUS after DBT and HHUS increases the benign biopsy rate.

## Data Availability

Authors agree to make data and materials supporting the results or analyses presented in this manuscript available upon reasonable request.

## Abbreviations

CC: Craniocaudal
DBT: Digital breast tomosynthesis
HHUS: Handheld ultrasound
MLO: Mediolateral oblique
3D ABUS: Three-dimensional automated breast ultrasound
